# Recovery of Stored Aerobic Granular Sludge and Its Contaminants Removal Efficiency under Different Operation Conditions

**DOI:** 10.1155/2013/168581

**Published:** 2013-09-11

**Authors:** Zhiwei Zhao, Shuo Wang, Wenxin Shi, Ji Li

**Affiliations:** ^1^State Key Laboratory of Urban Water Resource and Environment, Harbin Institute of Technology, Harbin 150090, China; ^2^School of Environment and Civil Engineering, Jiangnan University, Wuxi 214122, China

## Abstract

The quick recovery process of contaminants removal of aerobic granular sludge (AGS) is complex, and the influencing factors are still not clear. The effects of dissolved oxygen (DO, air intensive aeration rate), organic loading rate (OLR), and C/N on contaminants removal characteristics of AGS and subsequently long-term operation of AGS bioreactor were investigated in this study. DO had a major impact on the recovery of AGS. The granules reactivated at air intensive aeration rate of 100 L/h achieved better settling property and contaminants removal efficiency. Moreover, protein content in extracellular polymeric substance (EPS) was almost unchanged, which demonstrated that an aeration rate of 100 L/h was more suitable for maintaining the biomass and the structure of AGS. Higher OLR caused polysaccharides content increase in EPS, and unstable C/N resulted in the overgrowth of filamentous bacteria, which presented worse NH_4_^+^-N and PO_4_^3−^-P removal. Correspondingly, quick recovery of contaminants removal was accomplished in 12 days at the optimized operation conditions of aeration rate 100 L/h, OLR 4 g/L*·*d, and C/N 100 : 10, with COD, NH_4_^+^-N, and PO_4_^3−^-P removal efficiencies of 87.2%, 86.9%, and 86.5%, respectively. The renovation of AGS could be successfully utilized as the seed sludge for the rapid start-up of AGS bioreactor.

## 1. Introduction

Aerobic granular sludge (AGS) was considered to be a special kind of biofilm structure composing of self-immobilized cells [1, 2]. With regular shape, smooth surface, and compact and strong microbial structure, AGS has the advantages of better settling property, lower consuming, higher biomass retention, and treatment efficiency than normal activated sludge [3, 4]. Therefore, AGS has been proposed as a promising technology which could be widely applied in the treatment of high organic wastewater [5] and wastewater with toxic components [6] as well as wastewater with toxicity and heavy metal [7, 8]. AGS technology possesses the ability to contribute to and improve the biological treatment of wastewater. Compared to normal wastewater treatment plants, similar efficiencies at lower costs could be achieved with the compact AGS technology [9]. 

Microbial forces by extracellular polymeric substances (EPS) were regarded as the significant factor in the formation process of AGS [10]. Protein content in EPS, rather than polysaccharides, was enriched in AGS [11, 12] which was known as the key component of EPS. It is believed that high protein content and relatively high PN/PS (the ratio of protein content and polysaccharides content in EPS) ratio would be a good situation to keep stable internal microstructure and high biomass retention. Enrichment of EPS assisted granulation, enhanced granules stability, which is important during the operation of AGS bioreactor, and reduced the loss of granules in storage [1].

However, AGS would lose its microbial activity under extended idle conditions or after long-term storage, which is one of the main problems hindering the practical application of AGS technology [13]. In addition, the cultivation of AGS, quick start-up, and stable operation of AGS bioreactor restricted the development of AGS technology, from lab scale to pilot scale as well [12]. Consequently, the long-term storage and quick recovery of contaminants removal of AGS are remarkably important for its full-scale application. The storage of AGS, including the bioactivity preservation and physical characteristics maintenance, was influenced by DO, OLR, and C/N [1, 14]. Moreover, the rapid recovery of contaminants removal and subsequent quick start-up of AGS bioreactor were also controlled by such factors.

In order to improve the flexibility and enhance the practicability of AGS technology, this study investigated the effects of DO, OLR, and C/N on the settling property, EPS, and microbial activity of AGS, and further to explore optimal operation conditions on contaminants removal.

## 2. Materials and Methods

### 2.1. Aerobic Granular Sludge Cultivation

The experiment was carried out in a sequencing batch airlift reactor (SBAR). The working volume of the reactor is 5.4 L, with a height of 100 cm and an internal diameter of 10 cm. The internal riser pipeline was 70 cm in height, 6 cm in internal diameter, and 2 cm leaving the bottom. Compressed air was supplied via a diffuser at the bottom of the reactor with a flux of 120 L/h. Effluent was discharged from the middle port of the reactor with a volumetric exchange ratio of 50%. The operating cycle time was 6 h, including 30 min for idle, 30 min for static feeding, 5 min for settling, 5 min for effluent discharge, and the rest of the time for aeration. Accordingly, the temperature of the mixed liquid was kept at ambient temperature, and influent pH and solids retention time (SRT) were adjusted to 7.0–7.2 (by 1 mol/L HCl and 1 mol/L NaOH) and 30 days, respectively.

### 2.2. Synthetic Wastewater and Seed Sludge

The components and concentrations in synthetic wastewater were listed as (mg/L) NaAc 830.0, CaCl_2_ 60.0, MgSO_4_ 42.0, NH_4_Cl 240.0, EDTA 42.0, NaHCO_3_ 250.0, and K_2_HPO_4_ 58.0, KH_2_PO_4_ 24.0 [14], in element solution 1 mL, which gave a total COD concentration of 1200 mg/L; the concentration of NH_4_^+^-N was 60 mg/L, and the concentration of PO_4_^3−^-P with 16 mg/L. Trace element solution contained the following components (g/L): FeCl_3_·6H_2_O 1.5, H_3_BO_3_ 0.15, CuSO_4_·5H_2_O 0.03, KI 0.03, MnCl_2_·4H_2_O 0.12, Na_2_MoO_4_·2H_2_O 0.06, ZnSO_4_·7H_2_O 0.12, and CoCl_2_·6H_2_O 0.15 [15]. The seed activated sludge was taken from the aerobic tank of Wenchang wastewater treatment plant (WWTP in Harbin, China) with an anoxic/oxic process (A/O process).

### 2.3. Storage and Recovery of Aerobic Granular Sludge

The inoculated AGS had been stored at a 4°C refrigerator for more than 6 months and then regained in the previously mentioned SBAR after washing by distilled water for 3 times, with the same operation conditions and synthetic wastewater components in different reactors. The temperature of the mixed liquid was kept at ambient temperature, and influent pH was adjusted to 7.0–7.2. DO (Do concentration was represented by air intensive aeration rate, 50, 100, 150, and 200 L/h), OLR (2, 4, 8 and 16 g/L·d), and C/N (100 : 5, 100 : 10, and 100 : 20) were regulated and controlled to investigate the recovery on contaminants removal characteristics.

### 2.4. Analysis Methods

COD, NH_4_^+^-N, NO_2_^−^-N, NO_3_^−^-N, PO_4_^3−^-P, mixed liquor suspended solids (MLSS), and mixed liquor volatile suspended solids (MLVSS) were analyzed according to the Standard Method [16]. Granules size and wet density were determined according to the methods by Laguna et al. and Schwarzenbeck et al. [17, 18]. The microstructure and morphology of the AGS were observed by scanning electron microscope (SEM, S-4800N, Japan). Sludge volume index (SVI) was determined according to the settled bed volume after 30 min settling and the dry biomass weight [14]. The extraction of EPS was performed by the usage of ultrasound-formamide-NaOH method [1]. Total polysaccharides (PS) and total protein (PN) contents in EPS were quantified by Dubois et al. and Lowry et al. [19, 20].

## 3. Results and Discussion

### 3.1. Storage of Aerobic Granular Sludge

Mature AGS cultivated in the SBAR was kept at a 4°C refrigerator, and pH was adjusted to 7.0–7.2; especially, the feed liquid was replaced every two weeks. The morphology and integrity of AGS were both in good condition after 6 months of storage. The physical parameters were listed in Table 1. Granules size and wet density were basically unchanged after storage; however, AGS had apparent variation in its biomass and settling property. Biomass retention decreased from 7.73 to 7.25 mg/L, probably due to the release of soluble organic material and cell hydrolysis as reported by Tay et al. [21]. After 6 months of storage, its settling property deteriorated as shown from the values of SVI. SVI obviously increased from 49.3 to 64.1 mL/g, indicating that granules cannot maintain better settling property after long-term storage.

### 3.2. Recovery of Contaminants Removal Efficiency

AGS after the 6-month storage was utilized as the seed sludge for the quick start-up of the SBAR. Before the seeding, the granules taken from the 4°C refrigerator should be washed for three times to remove the fermentation products and the residual nutrient substances.

#### 3.2.1. DO (Air Intensive Aeration Rate) 

**Figure 1:**
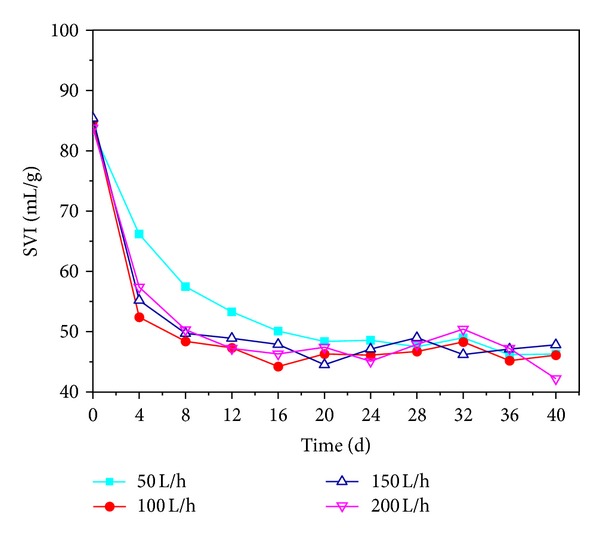
Variations of SVI at different air intensive aeration rates.

*Effects of DO on the Settling Property*. As shown in Figure 1, AGS had good settling property in the reactivation process. SVI of the granules decreased obviously in the first 8 days, and the granules had faster decline rate at the air intensive aeration rates of 100, 150 and 200 L/h than 50 L/h. The SVI of AGS decreased to 57.5, 48.4, 49.7, and 50.3 mL/g at the 8th day; then, it was maintained at a low level indicating their excellent settling ability. The granules revived under different air intensive aeration rates (100, 150, and 200 L/h) obtained quick recovery on its settle capacity. The SVI of granules reactivated at air intensive aeration rate of 100 L/h varied from 84.9 to 46.1 mL/g, which demonstrated that he granules had better settling property. In addition, relatively low air intensive aeration rate could be more economic and conductive for large-scale production and practical application. Therefore, 100 L/h aeration rate is good in the recovery of settling property.

**Figure 2:**
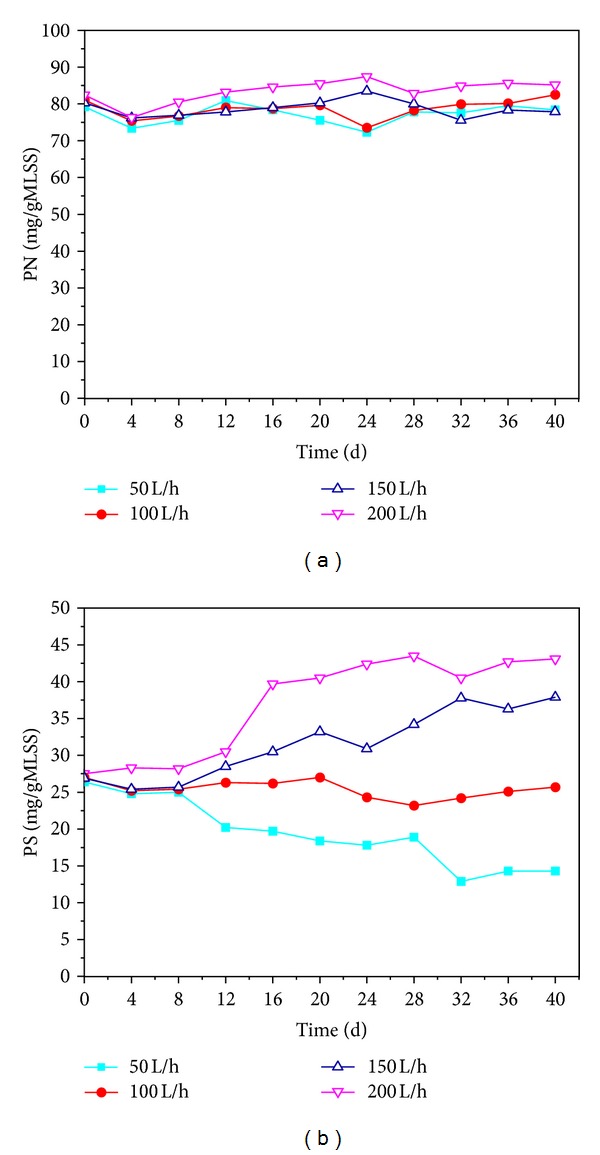
Variations of PN (a) and PS (b) contents in EPS at different air intensive aeration rates.

*Effects of DO on Granules Structure*. Bacterium can secrete sticky materials called EPS constituting proteins (PN), polysaccharides (PS), humic acids, and lipids, which could assist cell adhesion; thereby, it should be helpful to initiate the aerobic granulation process [12] (Schmidt et al., 2004). Protein and polysaccharides contents in EPS of AGS before the 6 months of storage were visually the same as those after the storage, with PN content of 80.3 mg/gMLSS and PS content of 27.0 mg/gMLSS. The ratio of PN/PS was 3.0. As illustrated in Figure 2(a), protein content was basically unchanged, implying the stable internal structure of AGS. PN content in EPS of the granules was 78.4, 82.5, 80.3, and 82.4 mg/gMLSS under different air intensive aeration rates (50, 100, 150, and 200 L/h). However, PS content under different air intensive aeration rates changed a lot (Figure 2(b)). PS content in EPS declined from 27.0 to 14.3 mg/gMLSS at air intensive aeration rate of 50 L/h, while it declined to 37.9 and 43.1 mg/gMLSS at air intensive aeration rate of 150 and 200 L/h. The variation of PS content caused unsteady PN/PS ratio and resulted in the fast disintegration of granules [13]. High PN content could be the cross-linked network by attraction of organic and inorganic materials [22] and the bridge of microbial cells once aerobic granules formed, which was consistent with the results of Adav et al. and Wang et al. [23, 24]. The present findings indicate that the induction of coaggregation and intracellular interaction by EPS played a significant role in the formation and maintenance of AGS.

**Figure 3:**
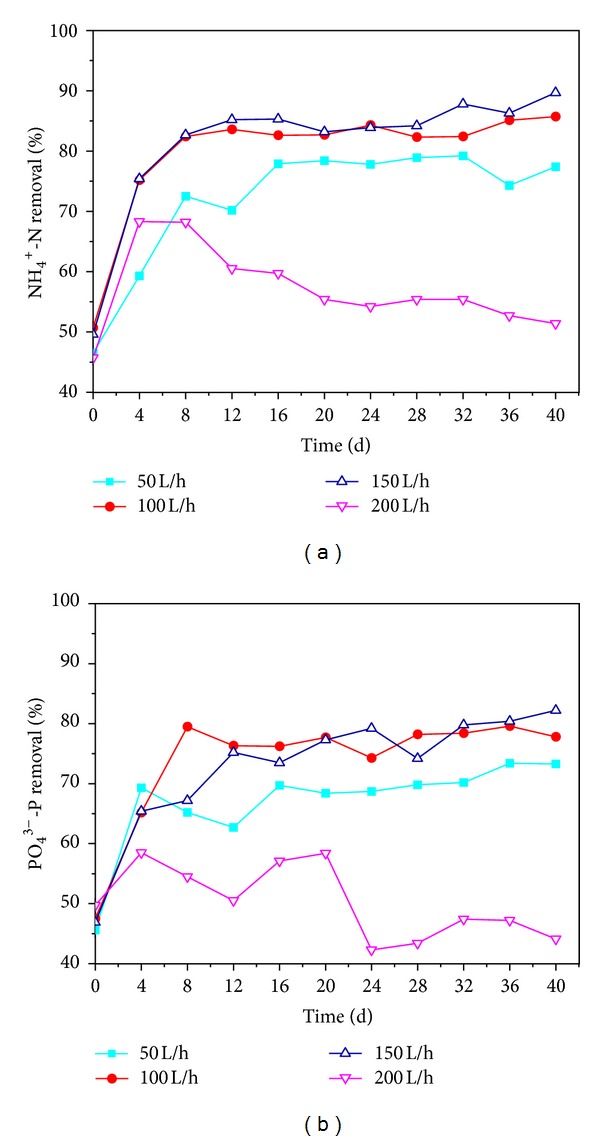
Variations of NH_4_^+^-N (a) and PO_4_^3−^-P (b) removal at different air intensive aeration rates.

*Effects of DO on Contaminants Removal*. DO was an important factor influencing nitrification and denitrification, which also expressed the key effect on the phosphorus release in anaerobic phase and the phosphorus uptake in aerobic phase [14]. Therefore, NH_4_^+^-N, and PO_4_^3−^-P removal efficiency would be greatly impacted by DO. Before the storage, AGS possessed good COD, NH_4_^+^-N and PO_4_^3−^-P removal performance, with COD, NH_4_^+^-N, and PO_4_^3−^-P removal efficiency of 90.2%, 93.5%, and 94.2%, respectively. However, the granules bioactivity to remove contaminants decreased after the storage. Microbial activity of aerobic granules began to revive, and NH_4_^+^-N removal ability was enhanced with the recovery progress (Figure 3(a)). As can be seen in the figure, NH_4_^+^-N removal rates were kept increasing in the first 8 days, especially for the air intensive aeration rates of 100 and 150 L/h. While for the air intensive aeration rate of 200 L/h, the story was somehow different, NH_4_^+^-N removal rate increased in the first 4 days and then decreased after 20 days. After 20 days reactivation, NH_4_^+^-N removal accomplished high removal efficiency of 77.4%, 85.7%, and 89.7% under aeration rates of 50, 100, and 150 L/h, which indicated that the granules gained better NH_4_^+^-N removal recovery performance. However, NH_4_^+^-N removal rate declined to 55.4% at the aeration rate of 200 L/h, probably because the sufficient oxygen supply led to the low bioactivity and slow specific growth rate of autotrophic bacteria including ammonia oxidized bacteria (AOB) and nitrite oxidized bacteria (NOB) [25, 26].

As displayed in Figure 3(b), the variations of PO_4_^3−^-P removal were basically the same and presented a good correlation with NH_4_^+^-N removal. PO_4_^3−^-P removal efficiency of the granules that recovered at aeration rate of 100 and 150 L/h increased up to 80% after activation for 16 days, which is higher than other recovery conditions. The granules had the characteristic of phosphorus accumulating potential with concomitant uptake of soluble organic carbon and release of phosphorus in the anaerobic stage, followed by rapid phosphorus uptake in the aerobic stage [27]. However, PO_4_^3−^-P removal of the granules that revived at an aeration rate of 200 L/h reached its maximum and then obviously declined to 47.2%. The result indicated that the sufficient oxygen supply might decrease the anaerobic zone inside AGS, which hindered the microbial activity of phosphorus accumulating organisms (PAO) and inhibited the phosphorus release and uptake process.

It is believed that the microstructure of the granules could be sustained and microbial activity retained good performance. The results showed that DO had a major impact on the settling property and contaminants removal efficiency of AGS. In addition, protein content in EPS was almost unchanged, which demonstrated that air intensive aeration rate of 100 L/h was more suitable for maintaining biomass and the structure of AGS.

#### 3.2.2. OLR

**Figure 4:**
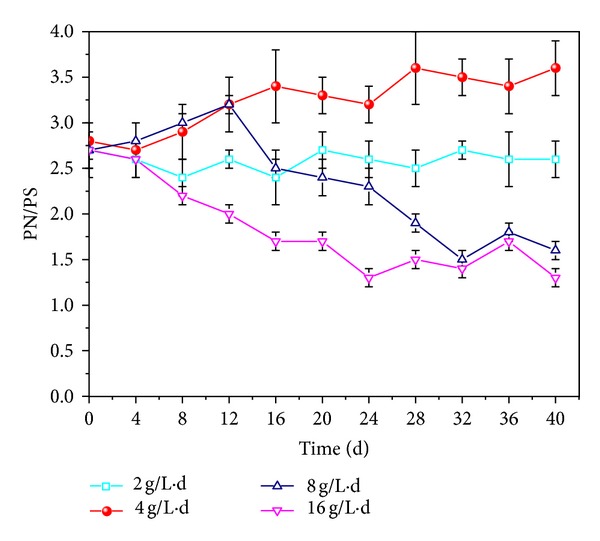
Variations of PN/PS ratio at different organic loading rates.

*Effects of OLR on Granules Structure*. It is believed that high protein content and relatively high PN/PS ratio would be good in keeping stable internal microstructure and high biomass retention [11, 12]. The variations of PN/PS ratio under different OLRs were illustrated in Figure 4. In the recovery process of AGS, PN and PS contents in EPS reactivated at 2 and 4 g/L·d were almost unchanged which possessed the stable structure of the granules. However, high OLR had a great impact on the PN/PS ratio. The variations of PN/PS revived at 8 and 16 g/L·d of OLR showed similar trend. PN content in EPS of AGS was stable, but PS content increased obviously, which were as high as 43.7 and 50.6 mg/gMLSS, resulting in the PN/PS ratio remaining at the low level of 1.5. Hence, relatively high PS content in EPS could reduce the integration and stability of the granules; however, Costerton et al. and Tay et al. discovered that high PS content was noted to facilitate cell-to-cell adhesion and strengthen the structure of granules through a polymeric matrix [28, 29].

*Effects of OLR on Contaminants Removal*. AGS did not lose all the contaminants removal abilities after the 6-month storage. The concentrations of contaminants in the effluent of the SBAR and contaminants removal efficiency after 31 days reactivation were listed in Table 2. COD removal efficiency reached about 80% under different organic loading rates in the first 8 days; then, COD removal was obtained at high efficiency of 89.2%, 89.4%, 90.5%, and 94.4%, respectively, which demonstrated good COD removal efficiency.   Nonetheless, NH_4_^+^-N and PO_4_^3−^-P removal was quite different; relatively high OLR conditions resulted in lower NH_4_^+^-N and PO_4_^3−^-P removal as a result of the disintegration and deterioration of AGS [13, 30]. The granules revived under OLR of 4 g/L*·*d gained good NH_4_^+^-N and PO_4_^3−^-P removal efficiency of 90.5% and 80.7%.

The granules reactivated at OLR of 4 g/L·d could maintain good structural integrity and high contaminants removal efficiency. Meanwhile along the recovery process, PS content in EPS was progressively increased which led to the disintegration of AGS and worse NH_4_^+^-N and PO_4_^3−^-P removal efficiency under higher OLR conditions. Therefore, AGS revived at OLR of 4 g/L·d was more suitable for the long-term stable operation of AGS.

#### 3.2.3. C/N

**Figure 5:**
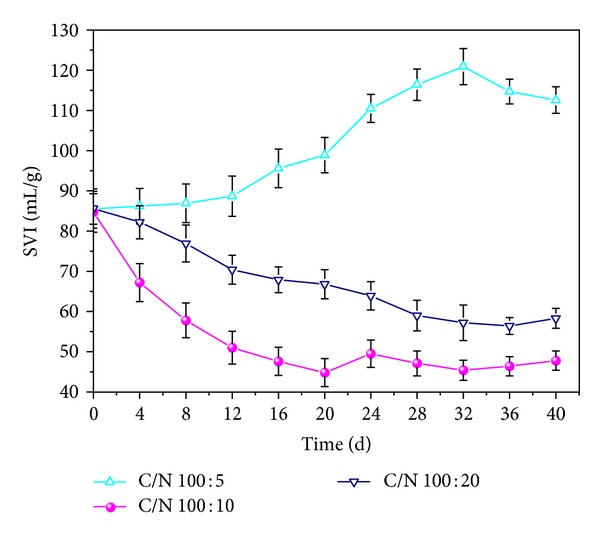
Variations of SVI at different C/N ratio.

*Effects of C/N Ratio on the Settling Property of AGS*. It is apparent in Figure 5 that low C/N caused good settling property in the recovery process. SVI values were 47.8 and 57.2 mL/g at C/N of 100 : 10 and 100 : 20, respectively. The SVI of the granules that revived at C/N of 100 : 10 decreased significantly faster than that of the granules revived at C/N of 100 : 20. However, the SVI of AGS reactivated at C/N of 100 : 5 obviously increased up to 110.5 mL/g and then was retained at a high level that presented worse settling ability. The finding revealed that the compact AGS grew in size but gradually lost the stability corresponding with the outgrowth of filamentous bacteria. Therefore, unstable C/N resulted in the overgrowth of filamentous bacteria, which presented worse contaminants removal efficiency [14, 31].

*Effects of C/N on Contaminants Removal Efficiency*. Effluent contaminants and contaminants removal efficiency after 31 days reactivation were listed in Table 2. Along with restoration, COD removal efficiency under different C/N conditions gained higher removal efficiency of 87.9%, 88.2%, and 89.6%, respectively, indicating excellent COD removal efficiency. Nevertheless, NH_4_^+^-N and PO_4_^3−^-P removal was quite distinct. NH_4_^+^-N removal efficiency at C/N of 100 : 5 was 62.4% because heterotrophic bacteria, whose growth rate was faster than those in a lower C/N, would be in a competitive advantage in inhibiting the activity of nitrifying bacteria and autotrophic bacteria [25]. Furthermore, PO_4_^3−^-P removal efficiency at C/N of 100 : 20 was 64.6% because of the competition of soluble organic carbon between PAOs and denitrifiers that hindered the microbial activity of PAOs [32]. The granules reactivated under C/N of 100 : 10 achieved both good NH_4_^+^-N and PO_4_^3−^-P removal efficiencies of 88.2% and 89.1%.

In this study, different C/N conditions had significant impacts on the settling property of AGS during the reactivation process. High C/N rate resulted in the overgrowth of filamentous bacteria, which presented worse NH_4_^+^-N and PO_4_^3−^-P removal efficiency. Hence, the granules that recovered at C/N of 100 : 10 were most stable with little variation on SVI and good NH_4_^+^-N as well as PO_4_^3−^-P removal efficiency after 12 days of reactivation.

### 3.3. Quick Recovery of Contaminants Removal

**Figure 6:**
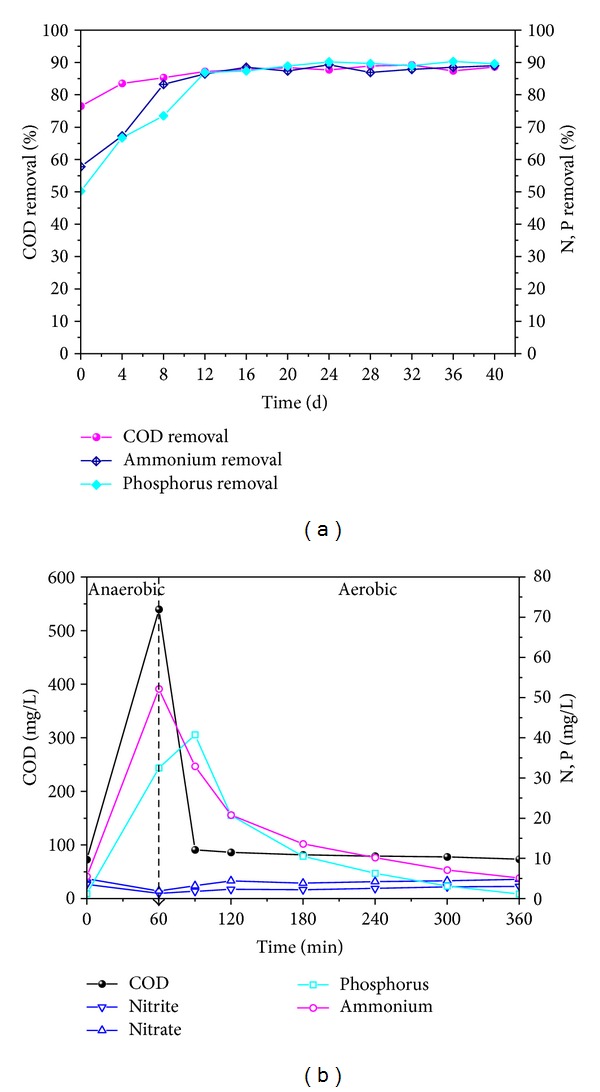
Contaminants removal under optimized operation conditions ((a) process; (b) cycle).

As displayed in Figure 6(a), the bioactivity of AGS progressively revived along with the recovery process under optimized operation conditions. According to the defined conditions previously mentioned, the optimal conditions were as follows: air intensive aeration rate 100 L/h, OLR 4 g/L·d, and C/N 100 : 10. Initially COD, NH_4_^+^-N, and PO_4_^3−^-P removal was low; however, with the reactivation progress, COD, NH_4_^+^-N, and PO_4_^3−^-P removal kept increasing. After 12 days of reactivation, AGS achieved the best recovery performance on microbial activity; COD, NH_4_^+^-N, and PO_4_^3−^-P removal efficiency could be quickly recovered to 87.2%, 86.9%, and 86.5%, respectively.

It is noticeable in Figure 6(b) that COD, NH_4_^+^-N, and PO_4_^3−^-P in effluent were 73.5, 5.1, and 1.1 mg/L in the stable cycle during the reactivation process, and the respective COD, NH_4_^+^-N, and PO_4_^3−^-P efficiencies were 86.4%, 90.2%, and 93.1%. Moreover, the nitrification and denitrification coefficiency was 75.2%, and the simultaneous nitrification and denitrification rate was 0.41 mmol/L·h. The results demonstrated that the granules revived under optimal operation conditions gained overall recovery performance. Furthermore, AGS technology could deal with a large number of conversion processes including COD-oxidation, ammonium oxidation, and biological phosphorus removal. The renovation of AGS in store could be successfully utilized as the seed sludge for the rapid start-up of AGS bioreactor.

## 4. Conclusions

The morphology and integrity of AGS were both in good condition after 6 months of storage. Dissolved oxygen had significant impacts on the recovery of the granules, and OLR and C/N had comparatively slight influence. 

The microstructure of the granules could be sustained; the microbial activity retained good performance; and protein content in EPS was almost unchanged, which demonstrated that air intensive aeration rate of 100 L/h was more suitable for maintaining its biomass and the structure of AGS. The granules reactivated at OLR of 4 g/L·d could maintain good structural integrity and high contaminants removal efficiency. PS content in EPS was progressively increased which led to the disintegration and worse NH_4_^+^-N and PO_4_^3−^-P removal efficiency under higher OLR conditions. Different C/N conditions had significant impacts on the settling property of AGS during the reactivation process. High C/N resulted in the overgrowth of filamentous bacteria, which presented worse NH_4_^+^-N and PO_4_^3−^-P removal efficiency. 

Correspondingly, quick recovery of contaminants removal was accomplished in 12 days at the optimal operation conditions of air intensive aeration rate 100 L/h, OLR 4 g/L·d, and C/N 100 : 10, in which COD, NH_4_^+^-N, and PO_4_^3−^-P removal efficiencies were 87.2%, 86.9% and 86.5%, respectively. The renovation of AGS in store could be successfully utilized as the seed sludge for simplifying the start-up and enhancing the long-term stable operation of AGS bioreactor.

## Figures and Tables

**Table 1:** Physical parameters of AGS before and after storage.

Physical parameters						
	SVI (mL/g)	MLSS (mg/L)	MLVSS (mg/L)	MLVSS/MLSS (%)	*ρ* (g/cm^3^)	Diameter (mm)
Before storage	49.3	8.45	7.73	91.5	1.040	2.8
After storage	64.1	8.45	7.25	85.8	1.032	2.6

**Table 2:** Effluent characteristics and contaminants removal under different operation conditions.

Effluent									
		COD (mg/L)	NH_4_^+^-N (mg/L)	NO_2_^−^-N (mg/L)	NO_3_^−^-N (mg/L)	PO_4_^3−^-P (mg/L)	COD^a^ (%)	NH_4_^+^-N^b^ (%)	PO_4_^3−^-P^c^ (%)
OLR (g/L·d)	2	56.0 ± 5.2	4.4 ± 0.3	13.3 ± 0.2	—	8.0 ± 0.4	89.2	89.3	52.5
	4	73.8 ± 6.9	4.4 ± 0.3	3.1 ± 0.2	—	3.3 ± 0.3	89.4	90.5	80.7
	8	107.8 ± 8.9	7.6 ± 0.4	3.5 ± 0.1	0.5 ± 0.3	4.1 ± 0.4	90.5	81.3	75.9
	16	120.0 ± 10.3	9.2 ± 0.4	4.2 ± 0.2	—	5.2 ± 0.3	94.4	77.0	68.9

C/N	100 : 5	63.0 ± 6.4	10.1 ± 0.3	1.8 ± 0.1	3.3 ± 0.1	4.7 ± 0.2	87.9	62.4	71.3
	100 : 10	58.0 ± 6.1	3.7 ± 0.2	1.9 ± 0.1	3.1 ± 0.1	4.5 ± 0.2	88.2	89.1	72.7
	100 : 20	57.5 ± 6.5	7.7 ± 0.3	2.9 ± 0.1	7.5 ± 0.5	5.8 ± 0.3	89.6	77.4	64.6

a: COD removal; b: NH_4_^+^-N removal; c: PO_4_^3−^-P removal; —: not detected.
